# Supplementary material for: Nereid as a regular satellite of Neptune
Source: Sci Adv. 2026 May 20;12(21):eaeb1429. doi: 10.1126/sciadv.aeb1429 (PMC13189092; doi:10.1126/sciadv.aeb1429)
Supplement: Supplementary file 1 — Supplementary Text Figs. S1 to S3 [file sciadv.aeb1429_sm.pdf]

Science Advances
AAAS

Supplementary Materials for

Nereid as a regular satellite of Neptune

Matthew Belyakov *et al.*

Corresponding author: Matthew Belyakov, mattbel@caltech.edu

Sci. Adv. **12**, eaeb1429 (2026)
DOI: 10.1126/sciadv.aeb1429

This PDF file includes:

Supplementary Text
Figs. S1 to S3

Other Simulation Outcomes

In the supplementary material, we show other simulation outcomes besides the one shown in Figure 3 of the main text, using the same plotting scheme. The most common outcome of our simulations as shown in Fig. S1 is Triton being ejected (or colliding with Neptune) similar to other works that have simulated the same process *(36)*. Typically, this process leaves several irregular satellites in orbit. In Fig. S2 we show an example of a simulation run where Triton circularizes and destroys the satellite system without having perturbed a moon to a Nereid-like orbit. We also observe simulations in which Triton perturbs several satellites to irregular orbits (Fig. S3). Occasionally, these moons also happen to acquire retrograde or relatively circular and distant orbits – irregular, though not necessarily Nereid-like.

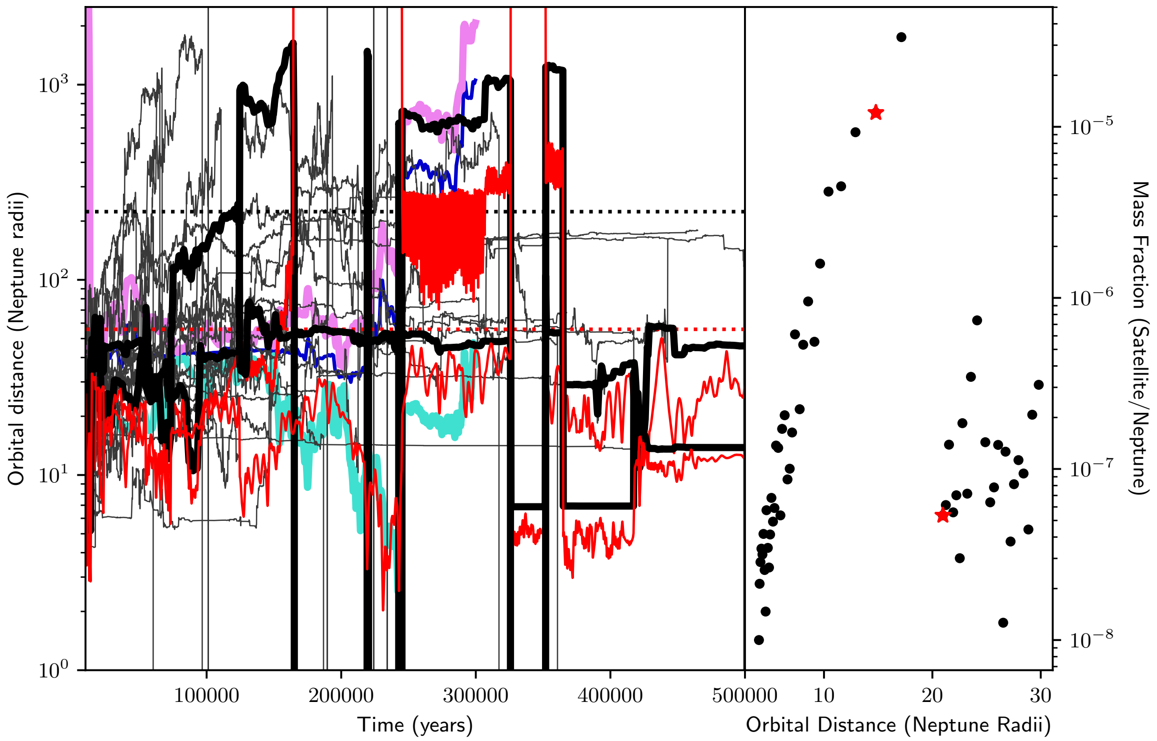

Figure S1: An example of a simulation where Triton is ejected from the system, leaving behind one satellite which lands on a low-eccentricity orbit, while one has significant inclination and eccentricity.

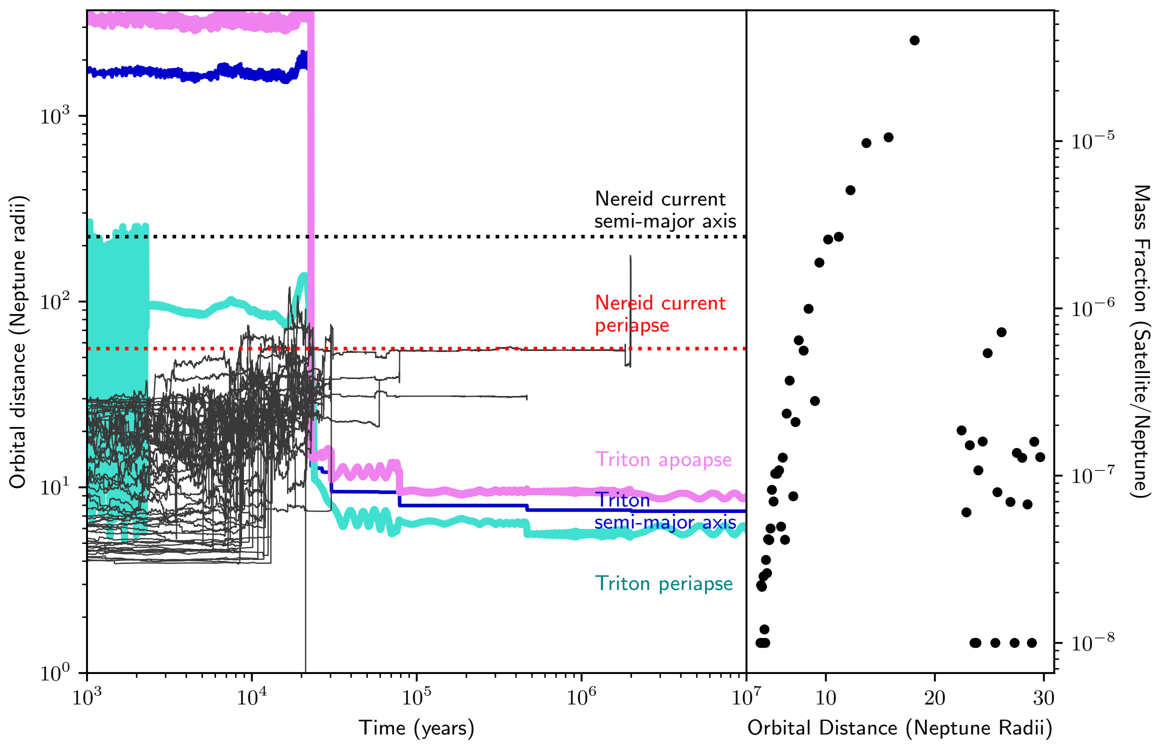

Figure S2: An example of an unsuccessful simulation run, where Triton circularizes and all of the satellites get ejected, or collide with each other, Triton, or Neptune, with the latter being the most common outcome.

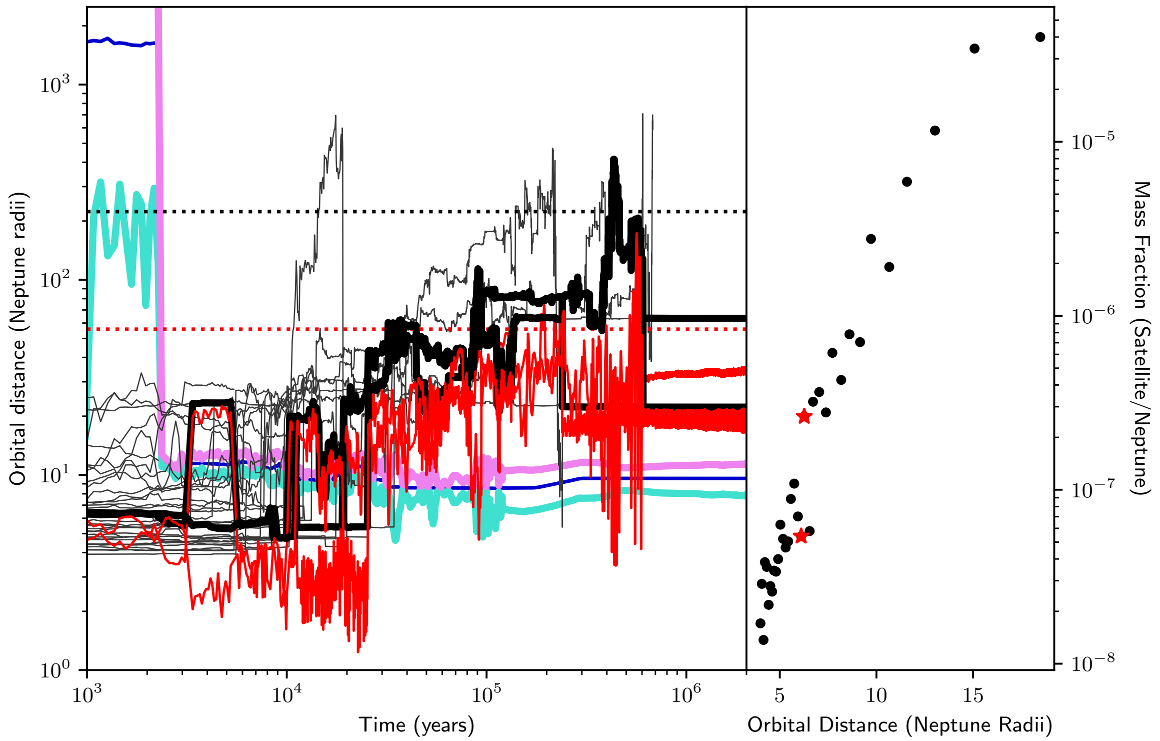

Figure S3: An example of a simulation where three objects successfully cross Triton's apoapse, one of which lands on a relatively low-eccentricity orbits, while one is closer to Nereid's orbit.
